# Diphtheria and Its Treatment

**Published:** 1864-05

**Authors:** F. R. Maillard

**Affiliations:** Beetown, Wis.


### DIPHTHERIA, AND ITS TREATMENT.

By F. R. MAILLARD, M. D., of Beetown, Wis.

*Messrs. Editors* :—A non-professional reader of almanacs and medical journals would imagine that nothing new could be written on the subject of Diphtheria. As proof that such is not the case, see a letter from C. D. Meigs, in the *American Journal of the Medical Sciences* for Jan., 1864, from which we of the West have gained some new light. Professional courtesy requires us to believe that Dr. Meigs describes Diphtheria as *he sees* it, and gives the treatment *he* has found most successful. If so, Diphtheria in Philadelphia, and Diphtheria in Wisconsin differ widely in symptoms, and in each locality demands an entirely different treatment. I propose to note a few of the symptoms as I have *felt* them, and as I have observed them for the last four years, and then give the treatment I have found the most successful. Of its origin and cause I shall say nothing, and but little of its nature.

Diphtheria, or Diphtheritic Fever, naturally divides itself into three stages, first, an intermittent; second, a continued; third, a typhoid stage. In the first stage, Diphtheria is as certainly a periodic disease as is miasmatic intermittent. The premonitory symptoms are those which belong to fever generally. The fever usually commences, and is always highest, near midnight, and there is generally a slighter exacerbation about noon. The febrile action may be slight, and hardly noticed, or severe, causing delirium. Sometimes there are three or four exacerbations before the patient will confess to any soreness of the throat, but then they are always slight. If the patient is capable of answering you understandingly, he will, almost always, refer his most acute pain to the articulation of the atlas with the cranium. In this stage, be the fever slight or severe, it is *sthenic*. It is said that, "in some cases it assumes the typhoid grade from the first." This is possibly so, but in my experience, which now extends to between two and two hundred cases, I am not absolutely certain that a single case *commenced* with that grade, though many cases when first seen, were decidedly *asthenic*.

In the second, or continued stage, the fever is more or less remittent, and may be decidedly sthenic, and is always accompanied by soreness of the throat, swelling of the glands about the angle of the jaw, and the peculiar exudation or deposit on the fauces.

The third, or typhoid stage, has been so well and often described that it is useless to try to better it. The peculiar exudation on the fauces is not more characteristic of Diphtheria, than is the condition of the secretory organs generally. The urine is decreased in amount, and often albuminous. The bile is always vitiated, and the liver generally sluggish, and occasionally in the later stage enlarged, as I have verified by post-mortem observation. That it is not a local disease is proven by the fact that general, always precedes the local symptoms.

Let us, now, lay aside all ideas of a relationship between Diphtheria and Scarlatina, or Rubeola, and see what we are to do for a case of this disease. The first question is, can we arrest it, or will it run a certain course, and then terminate of itself? There is no doubt that some patients will recover without any treatment, and even in spite of bad treatment. But it is equally true, that some will die, treat them as you will. If we assume that the patient cannot be cured, then our only course is to sustain him, and watch the fight. In what I have denominated the first and second stages, it may, in the vast majority of cases be arrested, and that almost as suddenly and as certainly, as intermittent from miasmatic causes. The first case of Diphtheria that I ever treated, or ever saw, occurred in myself. Here was a splendid chance to experiment, and I used it. I was satisfied that I required a powerful alterative. Authorities said "they must not be used. The disease is asthenic. The treatment is, *caustics* and *stimulants* locally; *stimulants* and *tonics* internally." Well, I run the gauntlet of caustics, from burnt alum to caustic potash, and of local stimulants, from sol. of chlor. potassa to a strong sol. of nit. arg. Internally, pot. chlor. quinine, tinct. ferri chlor. and whiskey punch, with an occasional opiate to procure rest. In spite of it all the disease extended to the posterior nares, and I became convinced that something must be done.

I therefore, threw aside everything *but the punch*, and as I was too weak (as I thought) to stand a course of mercury, ordered a sol. of iodide potassa, ʒj of the salt to a fl ℥j of water. Of this I took ʒj every four hours, using the same as a gargle, and injecting it into the nares. At night took gr. viij of dover's powder, and slept for a few hours, when I was awakened struggling for breath. I seized a pair of curved forceps and literally tore a passage for breath. This I repeated two or three times a day for some days, using nothing but the whiskey punch, iodide potassa and opium. Convalescence was slow, and the Schneiderian membrane has never *fully* recovered.

This experience effectually *cured* me from withholding alteratives when indicated. In the first stage the object should be to prevent its passing to the second. This we shall accomplish by overcoming the torpidity of the secretory system, and exciting the excretory to an equal degree, while at the same time we endeavor to give tone to the system of organic life. If the attack is severe, nature often gives us a hint as to the first step to be taken. She produces emesis. If she has not done this, and the patient is of good constitution, I give a full dose of ipecac, and as soon as convenient, follow by sub. mur. hg. gr. v. An hour after give a pill composed of sub. mur. hg., gr. j; ext. belladonna, gr. ⅛; and repeat every hour till it operates freely as a cathartic, or the specific effects of the belladonna are induced, when they are discontinued, and sulp. quinine given, in doses sufficiently large and often, to induce its specific effect, before the time for the next exacerbation of the fever. Patients usually want something done locally, and of all the caustics, washes, and gargles that I have used, none has satisfied me as well as a gargle of equal parts of water and tinct. guiac, used several times a day. If this does not check the advances of the deposit, the same course is pursued the next day, only omitting the emetic, when in the vast majority of cases the patient is dismissed.

The second stage does not last long, hence, what you do, must be done quickly. The indications are not materially different. The same treatment, (omitting the emetic,) is very often all that is required to check the fever, and arrest the deposit on the fauces. I seldom repeat the mercurial the third time, but if anything more is needed give quinine in solution with tinct. ferri chlor. If there is much excitement of the nervous system give tinct. belladonna in sol. of pot. chlorat., always insisting on the patient taking sufficient nourishment.

In the third stage, if the mercurial is given it must be with caution, and the powers of life sustained by every means at our command.

This is an outline of the treatment I have found most generally successful. But no general rules will apply to each particular case, hence the intelligent physician will always, from the general, deduce the particular rules, applicable to each individual case. If the physician will bear in mind that he has to deal with a disease, essentially periodic in its nature, he will hardly err in giving the quinine. The amount of the belladonna sometimes has to be varied several times in the same case, and occasionally cannot be borne at all. The same is true of the mercurial. If it increases the nervous sensibility, or excites the pulse, discontinue it immediately. Since it has become so decidedly fashionable to mention *calomel* only to curse it, you will perhaps allow me to aver that when the object is to influence the secretory functions with promptness and certainty, the Materia Medica has as yet, furnished me nothing equal to the mercurials. The reason why I prefer belladonna to any other narcotic, is that it will as generally quiet the nervous symptoms, as any of them, and it acts upon the whole excretory system, while it in a great degree counteracts the tendency of the mercurials to produce salivation. Though this is not a local disease, local treatment is not unimportant, for it is the absorption of vitiated secretion from the diseased surface, that causes the typhoid symptoms of its later stage. When no local treatment is used, we occasionally see the fever abate, and the false membrane nearly all thrown off, but a few patches remain and so does the peculiar sickening odor of the breath. As long as this remains, the patient is not free from danger. When in this stationary condition the guaiac gargle will, often in a few hours, clear the throat, and the odor at the same time disappears.

Another advantage it has seemed to me to possess, viz., preventing that most troublesome sequela, paralysis of the parts concerned in deglutition and speech. Under every other local application that I have used, paralysis, more or less complete, has occasionally occurred but never when the guaiac has been faithfully employed.

If what I have written should induce even a few of the readers of the *Journal* to *observe* Diphtheria for *themselves*, (and publish their observations, too,) regardless of the almost interminable mass of dogmatism which has been promulgated by American as well as European writers, I shall have accomplished all that I wished, and this truly fearful disease will not prove as fatal in their practice as it has in the country generally, for the past few years.

